# Monitoring Biochemical Changes of Neuroblastoma Cells in Early Stages After X-Ray Exposure by Using Fourier-Transform Infrared Spectroscopy

**DOI:** 10.3390/s24237459

**Published:** 2024-11-22

**Authors:** Rosario Esposito, Marianna Portaccio, Roberta Meschini, Ines Delfino, Maria Lepore

**Affiliations:** 1 Dipartimento di Ingegneria Chimica, dei Materiali e della Produzione Industriale, Università di Napoli “Federico II”, 80125 Napoli, Italy; rosario.esposito2@unina.it; 2 Dipartimento di Medicina Sperimentale, Università della Campania “Luigi Vanvitelli”, 80138 Napoli, Italy; marianna.portaccio@unicampania.it (M.P.); maria.lepore@unicampania.it (M.L.); 3 Dipartimento di Scienze Ecologiche e Biologiche, Università della Tuscia, 01100 Viterbo, Italy; meschini@unitus.it; 4 INAF-Osservatorio Astronomico di Capodimonte Napoli, Salita Moiariello 16, 80131 Napoli, Italy

**Keywords:** neuroblastoma cells, ionizing radiation, FTIR microspectroscopy, DNA, protein and lipid changes

## Abstract

X-ray radiation treatments are largely adopted in radiotherapy, and Fourier-transform infrared microspectroscopy (μ-FTIR) has already been demonstrated to be a useful instrument for monitoring radiotherapy effects. Previous works in this field have focused on studying the changes occurring in cells when they are fixed immediately after the irradiation or 24 and 48 h later. In the present paper, changes occurring in SH-SY5Y neuroblastoma cells in the first hours after the irradiation are examined to obtain information on the processes taking place in this not-yet-investigated time window by using μ-FTIR. For this purpose, cell samples were fixed immediately after X-ray exposure, and 2 and 4 h after irradiation and investigated along with unexposed cells. Different data analysis procedures were implemented to estimate the changes in lipid, protein, and DNA spectral contributions. The present investigation on the effects of X-ray in the first hours after the exposure is helpful for better describing the processes occurring in this time window that offer the possibility of a timely check on the efficacy of X-ray treatments and can potentially be applied for planning personalized treatment as required by the most advanced medical therapy.

## 1. Introduction

The study of the effects of exposure of biological macromolecules to ionizing radiation is a challenging task. Different physicochemical reactions can occur, and the subsequent interactions with nuclear DNA, nuclear and cell membranes, and cytoplasm induce a series of biomolecular changes. Radiation-induced DNA lesions, such as base deletions and oxidation, single- and double-strand breaks, and clustered damage sites, are considered critical to cell survival, and their relevance depends on dose, dose rate, and radiation energy transfer modalities. Less is known about the ionizing-radiation-induced effects on the other cellular components, namely, lipids, proteins, and carbohydrates. After the occurrence of DNA damage, complex repair mechanisms can be activated by the cells for facing radiation exposure [1,2,3], in which lipids, proteins, and carbohydrates are nowadays known to play a relevant role.

Fourier-transform infrared spectroscopy is nowadays considered a valuable tool for investigating biochemical changes occurring in cells during interactions with external agents [4,5,6,7,8,9]. Fourier-transform infrared microspectroscopy (μ-FTIR) has been demonstrated to be sensitive to molecular changes occurring in cells and tissues after exposure to ionizing radiation [10,11,12,13,14,15,16,17,18,19].

FTIR spectroscopy investigations on the changes induced by ionizing radiation in different cellular systems evidenced modifications in the position and intensity of the modes for CH_3_ symmetric stretching related to alterations of membrane fluidity [17,20]; shifts towards higher wavenumber values in the PO_2_ DNA-related stretching mode attributed to changes in the DNA conformation [17,21]; shifts of Amide II mode ascribed to changes of enzymes participating in DNA repair; modifications in the secondary structure of proteins [15,22]; variations in the ratio between the absorbance bands of CH_3_ and CH_2_ asymmetric stretching associated with the occurrence of an apoptotic process [23,24,25]; damages in the primary, secondary, and tertiary structures of nucleic acid [26]. In addition, the occurrence of cell recovery processes has been evidenced by the comparison between FTIR data related to cell samples fixed immediately or some hours after the irradiation. This evidence has been observed while studying the changes occurring in cells fixed immediately after the irradiation or 24 and 48 h later [17,18,27]. However, previous studies performed by using biological assays also indicated that the examination of the processes occurring in the first hours after X-ray exposure can be interesting [28,29]. These studies concern the effects induced on DNA, so no information on the other cellular components is given. Conversely, FTIR spectroscopy allows the complete monitoring of changes induced in DNA, protein, lipid, and carbohydrate components. For this reason, the present paper aims to investigate changes taking place in the first hours (2 and 4 h) after the irradiation by using μ-FTIR spectroscopy. This study is, to the best of our knowledge, the first study performed using this approach on the early (within the first 4 h after irradiation) effects of X-ray irradiation on cells. It is also designed to confirm the ability of μ-FTIR of sensing the effects of irradiation on cells. The SH-SY5Y cell line was used in this study because of its relevance in studying tumoral cell behavior and in neuroprotection studies for developing new medical procedures for preventing and treating central nervous system disorders [30,31,32,33,34,35].

## 2. Materials and Methods

### 2.1. Materials

DMEM medium, fetal bovine serum, penicillin, streptomycin, L-glutamine, and formaldehyde were provided by Sigma-Aldrich Co. (St. Louis, MO, USA) and used without any further treatments. SH-SY5Y (American Type Culture Collection, Manassas, VA, USA) is a human cell line subcloned from a bone marrow biopsy taken from a four-year-old female with neuroblastoma.

### 2.2. Sample Preparation and Treatment

SH-SY5Y cells were cultured in vitro in DMEM medium, supplemented with 15% fetal bovine serum, 1% penicillin, 1% streptomycin, and 1% L-glutamine. They were grown at 37 °C, 5% CO_2_, in 25 cm^2^ flasks. The cells were seeded on MirrIR slides (25 × 25 mm^2^) (Kevley Technologies, Chesterland, OH, USA), a specific reflection FTIR spectroscopy microscope slide, nested into Petri dishes (60 mm diameter). The protocol was meant to obtain, for each MirrIR slide, a number of cells (~4 × 10^5^), since in these conditions, cells were not confluent, with sufficient intercellular spaces for measurement of the background signal being available.

X-ray irradiation was performed on ice, using a Gilardoni MGL 200/8D machine (Gilardoni, Rome, Italy) operating at 250 kVp and 6 mA (dose rate 60 cGy/min). The cells were exposed to X-rays at two doses (2, 4 Gy) and then investigated along with unexposed cells (0 Gy). Immediately after X-ray exposure (t_0_ cells) and 2 and 4 h later (t_2_ cells and t_4_ cells, respectively), the cells were fixed in a 3.7% formaldehyde in PBS solution for 20 min at room temperature and then briefly washed in distilled water for 3 s to remove the PBS residue from the surface of the cells. The samples were then dried under ambient conditions and stored in a desiccator until spectral analysis.

### 2.3. μ-FTIR Spectroscopy

#### 2.3.1. Spectra Acquisition

Infrared absorption spectra of the cell samples were acquired at room temperature, using a Spectrum One FTIR (PerkinElmer, Shelton, CT, USA) spectrometer equipped with a Perkin Elmer Multiscope system infrared microscope and an MCT (mercury cadmium telluride) FPA (focal-plane-array) detector. The spectra were collected with an aperture of 100 × 100 μm^2^ in transflection mode.

The background signal was acquired in a region of the slide free of cells. Different regions of every slide were examined, and multiple spectra were acquired for each of the considered positions. All the spectra were collected with a spectral resolution of 4 cm^−1^ in the 4000–600 cm^−1^ spectral region using 64 scans and a 5 s acquisition time for each spectrum at room temperature.

#### 2.3.2. Data Analysis

##### Preliminary Analysis

The spectra acquired from SH-SY5Y cells were preliminarily treated by subtracting the corresponding background spectrum and operating a piecewise baseline correction [36]. The spectra were then elaborated using a vector normalization procedure to have comparable intensities adopting a standard normal variate (SNV) method [36,37].

##### Spectra Deconvolution Procedure

In the field of IR spectroscopy, the absorption spectra of large biological macromolecules often exhibit broad and overlapping peaks. This spectral congestion poses a challenge to interpreting the data accurately. One technique commonly employed to aid data interpretation is curve fitting, where the experimental spectrum is modeled as a sum of individual spectral contributions. However, the choice of the lineshape to be used to model the single component spectra significantly impacts the outcome of the curve fitting procedure.

Symmetric and asymmetric functions are commonly used lineshapes in curve fitting [38,39]. Symmetric lineshapes, such as Gaussian or Lorentzian functions, assume that the spectral peak is perfectly symmetrical around its center. The Gaussian function is given by the following:

(1) $$G\left(\nu \right)=\;\frac{A}{\sigma }\;\sqrt{\frac{4\;\ln2}{\pi }}\exp\left(-4\;\ln2\frac{{\left(\nu \;-\;\nu_{0}\right)}^{2}}{\sigma^{2}}\right)$$

Here, A represents the amplitude of the peak, ν_0_ is the peak center, and σ is the full width at half maximum. The Lorentzian function, on the other hand, is given by the following:

(2) $$L\left(\nu \right)=\;\frac{\frac{2A\sigma }{\pi }}{4{\left(\nu -\nu_{0}\right)}^{2}+\;\sigma^{2}}$$

The parameters are defined as for Equation (1). The problem with symmetric lineshapes arises when the experimental spectra exhibit asymmetry or skewed peaks. In such cases, performing the fit using symmetric lineshapes may result in poor data representation, leading to an inaccurate parameter estimation. To address this issue, modifications to well-known symmetric lineshapes were introduced to provide a certain degree of asymmetry. One widely used asymmetric lineshape is the pseudo-Voigt profile. It combines the characteristics of the Gaussian and Lorentzian functions to create a versatile lineshape. The pseudo-Voigt function is given by the following:

(3) $$f\left(\nu \right)=\alpha \;L\left(\nu \right)+\left(1\;-\;\alpha \right)G\left(\nu \right)$$

In this equation, α represents the mixing parameter that determines the relative contributions of the Gaussian and Lorentzian components. By adjusting the value of α, one can control the degree of asymmetry in the peak. The pseudo-Voigt profile offers a flexible approach to model asymmetric peaks, allowing a better fit to experimental data with broad and overlapping features. It strikes a balance between the Gaussian and Lorentzian functions, accommodating both symmetrical and asymmetrical spectral shapes.

The FTIR spectra were analyzed using pseudo-Voigt functions in a custom MATLAB code for the fitting procedure (Version 7.6, MathWorks Inc., Natick, MA, USA). Before the fitting process, a smoothing technique was applied to the data using a Savitzky–Golay finite impulse response (FIR) smoothing filter. In this case, a polynomial order of 2 and a frame length of 12 were used. The MATLAB routine “sgolayfilt” was utilized for this smoothing step. After the data were smoothed, the peak fitting was performed using the pseudo-Voigt profile. Certain parameters such as peak center and peak width, were initially fixed at the initial guess and then released. This approach helps to improve the accuracy and efficiency of the fitting process by providing a good starting point for the optimization algorithm. The fitting procedure considered a large range of the FTIR spectra, ranging from 3600 cm^−1^ to 800 cm^−1^. A total of 42 pseudo-Voigt profiles were simultaneously fitted to the data. This simultaneous fitting approach allows the consideration of multiple spectral features and their interactions, capturing the complexity of the observed spectral congestion.

##### Difference Absorbance Spectra

When differences between infrared spectra related to samples in various experimental conditions need to be evidenced, the difference spectra can be evaluated, as suggested by numerous studies in the literature [40,41,42,43], where the analysis of difference spectra allowed the authors to outline subtle differences between couples of FTIR spectra (difference spectrum = spectrum 1 − spectrum 2). The use of difference spectra helps us to directly compare two spectra taken from samples that underwent two treatments (i.e., 1 and 2). This enables the assignment of the outlined differences to the different treatments of the two samples, with a positive feature in the difference spectrum representing an intensity of the specific peak higher in spectrum 1 than in spectrum 2, and a negative feature representing the opposite. In the present case, difference spectra were obtained by subtracting the average SNV absorbance spectra for the different examined doses. For each of the three different fixation times, we examined two difference spectra, obtained by performing a point-by-point subtraction between specific average SNV absorbance spectra. The first spectrum considered is the difference spectrum evaluated by subtracting the average spectrum of control samples from the analogous of 2 Gy-irradiated samples (that we called d_ti_(2 Gy − 0 Gy) = difference spectrum obtained by subtracting the control spectrum from the 2 Gy dose spectrum, t_i_ cells, i indicating the time of fixation, hence, I = 0, 2, and 4). The second difference spectrum considered was obtained by subtracting the average spectrum obtained for control samples from the analogous for 4 Gy-irradiated samples (that we called d_ti_(4 Gy − 0 Gy) = difference spectrum obtained by subtracting control spectrum to the 4 Gy dose spectrum, t_i_ cells, where i = 0, 2, and 4).

##### Ratio Analysis

As is evident from the literature, a great effort has been made to identify biomarkers from infrared spectroscopy measurements. The ratios between the absorbance values or areas of selected peaks have been shown to play a potential role in this framework [4,17,18,19,24,44,45,46]. Concerning μ-FTIR applications to radiobiology, previous papers [17,18,19,44,45] indicated that useful information can be obtained using some specific ratios. In the present case, the ratios reported in Refs. [17,18,45] were evaluated by considering the absorbance values of the peak of interest.

## 3. Results and Discussion

### 3.1. Features of Infrared Spectra from Control Cells

**Figure 1:**
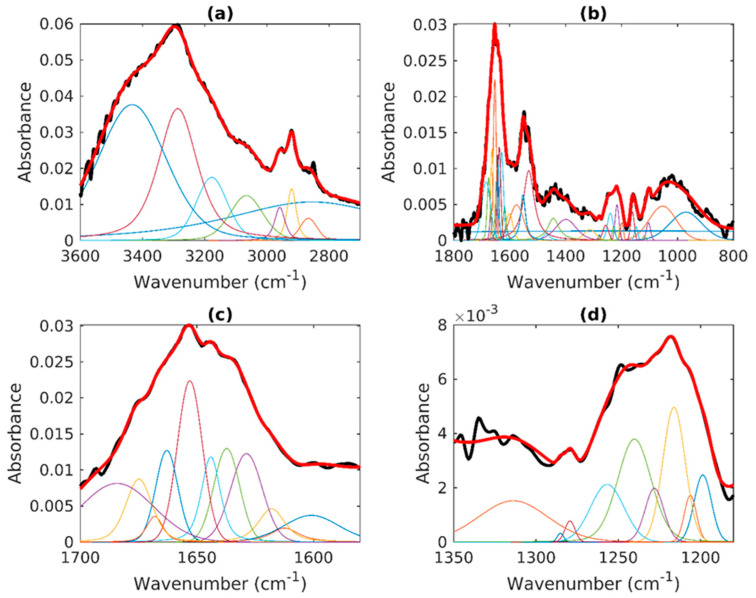
Average spectra of not-exposed cells and corresponding deconvolution in different spectral regions: (**a**) high-wavenumber region (3600–2600 cm^−1^), (**b**) fingerprint region (1800–800 cm^−1^), (**c**) Amide I region (1740–1580 cm^−1^), and (**d**) Amide III region (1350–1180 cm^−1^). The black and red lines indicate, respectively, experimental and reconstructed spectra. The other colored curves indicate the contributions of the different vibrational modes listed in Table S1 and their assignments.

In Figure 1, the average spectrum of a sample not exposed to X-ray is reported for the 3600–800 cm^−1^ spectral region that can be divided into two main zones. The range from 3600 to 2600 cm^−1^ (Figure 1a) is usually denoted as a high-wavenumber region (HWR) where contributions of proteins, lipids, and carbohydrates can be evidenced. The features located at 3286 and 3069 cm^−1^ are, respectively, attributed to the amide A and Amide B stretching motion of peptide backbones of proteins amino acids and O–H stretching of carbohydrate polysaccharides, while the contribution at ≈3178 cm^−1^ is assigned to the –NH_3_^+^ asymmetric stretching of free amino acids. The two peaks at ≈2954 cm^−1^ and ≈2866 cm^−1^ are attributed to the asymmetric and symmetric stretching of the methyl groups (–CH_3_), respectively, given by cellular proteins and lipids contribution. The structures at ≈2929 cm^−1^ and ≈2846 cm^−1^ are due to the asymmetric and symmetric stretching of the methylene groups of membrane lipids (–CH_2_), respectively. In the 1800–800 cm^−1^ region (the so-called fingerprint region) (Figure 1b), various peaks that are representative of proteins and nucleic acids are clearly observed. The two peaks at ≈1652 cm^−1^ and ≈1539 cm^−1^ are mainly assigned to the Amide I (C=O and C–N) and Amide II (N–H and C–N). The band at ≈1453 cm^−1^ is related to symmetric and asymmetric bending of the methylene and methyl groups (–CH_2_ and –CH_3_) and to –CH_2_ scissoring of proteins and lipids, and the peak at ≈1400 cm^−1^ is due to COO− group asymmetric stretching of proteins. The band at ≈ 1256 cm^−1^ is attributed to the Amide III structure. The asymmetric and symmetric PO_2_ stretching vibrations of the phosphodiester nucleic acid backbone are associated with the two bands at ≈1242 cm^−1^ and ≈1082 cm^−1^, respectively, with a contribution from C–O–P stretching of protein and lipids. All the other features present in the spectrum are reported in Table S1 together with their assignments [21,47,48]. The Amide I band, mentioned earlier and reported in Figure 1c, can be considered as a convolution of the contributions arising from the various secondary structures of proteins. The bands at 1636 cm^−1^ and 1624 cm^−1^ are attributed to parallel β-sheet subcomponent, and the 1614 and 1694 cm^−1^ band are related to the antiparallel β-sheet subcomponent.

The features at 1661 and 1652 cm^−1^ are attributed to α-helix secondary structures; β-turn structures contribute at the 1679 and 1674 cm^−1^ range; and unordered components are present at 1644 cm^−1^. The Amide III band, shown in Figure 1d, can also give interesting information about the secondary protein structure [17,47,49]. The components located at 1314 and 1294 cm^−1^ are generally attributed to the α-helix secondary structure. The structures at 1281 and 1256 cm^−1^ are assigned to random coil, while those positioned in the 1180–1240 cm^−1^ region can be considered as due to β-sheets [50,51]. The Amide I and II subcomponents are summarized in Table S1.

### 3.2. Analysis of Infrared Spectra from Cells Fixed Immediately After Irradiation (t_0_ Cells)

**Figure 2:**
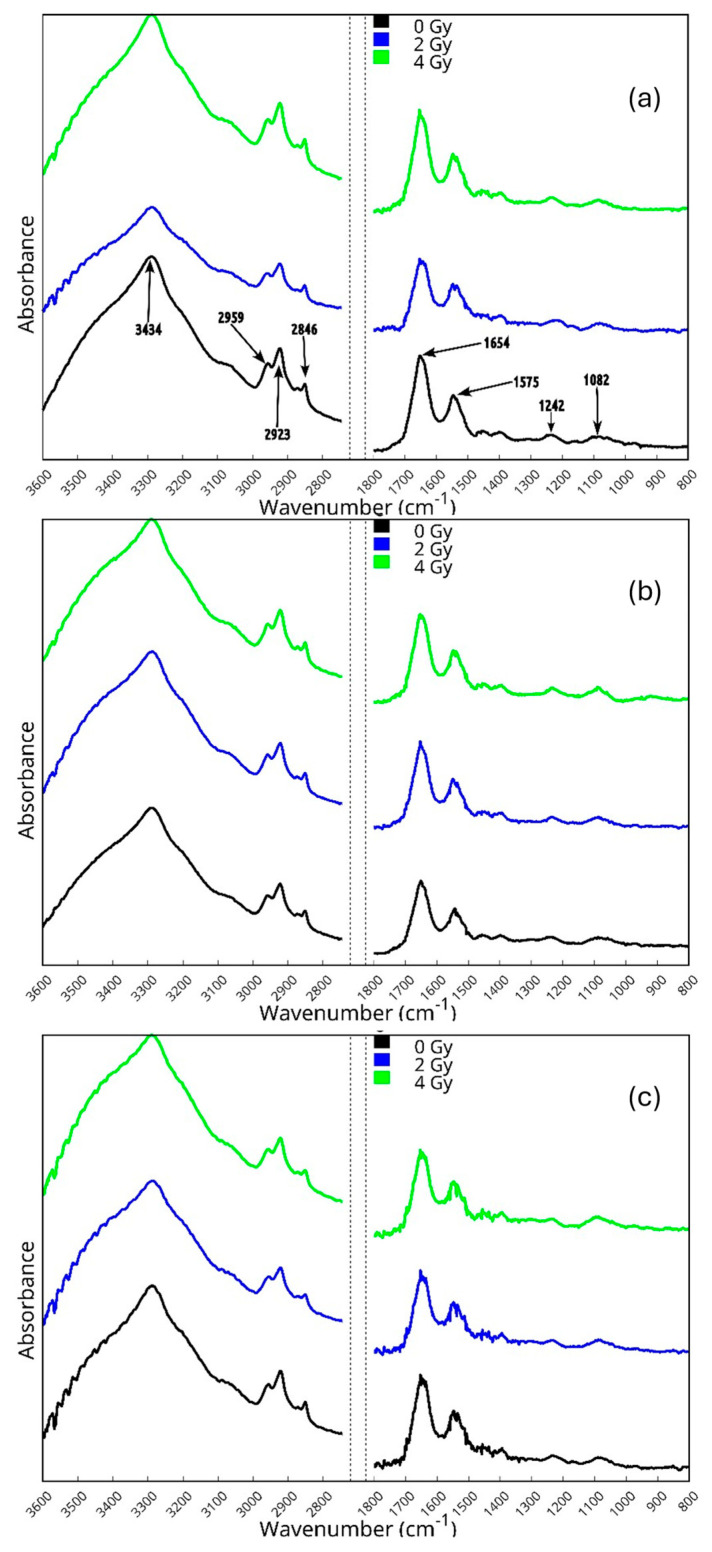
Average spectra for control and irradiated cells in the wavenumber region from 3600 to 800 cm^−1^ at different X-ray doses (0, 2, and 4 Gy) and fixed at different times after irradiation ((**a**) immediately after (t = 0), (**b**) 2 h after, and (**c**) 4 h after irradiation). The spectra are vertically shifted for avoiding overlapping. In panel (**a**), the spectral positions of relevant bands are indicated.

The average spectra for cells exposed to different doses of X-rays (0, 2, and 4 Gy dose) and fixed immediately after, 2 h after, and 4 h after the irradiation are reported in Figure 2. X-ray exposure induces wavenumber shifts and changes in the intensity and shape of the absorbance bands related to the lipid, protein, DNA, and carbohydrate cell components that can be outlined when comparing the irradiated sample spectra to the control one. For the here-investigated cell lines, the modifications occurring are not immediately evident, as noticed in previous works [16,17,45], and appropriate methods, such as those described in Section 2.3, should be adopted to highlight the occurring changes.

In Figure 2a, the average spectra for cells exposed to different doses of X-rays (0, 2, and 4 Gy dose) and fixed immediately after irradiation (t_0_ cells) are shown. In this figure, the spectral position of relevant bands that can be recognized by a visual inspection are indicated for the black curve related to the unexposed samples. The deconvolution procedure described above can evidence the wavenumber shifts for the different vibrational modes present in the spectra. In Table S2, the positions of the peaks observed for the various samples are reported; shifts in position with respect to the corresponding peak observed in the control sample higher than the spectral resolution of our experimental apparatus are reported in bold character. The most relevant changes for the high-wavenumber region are those related to CH_2_ and CH_3_ functional groups. The peak at around 2954 cm^−1^ assigned to CH_3_ asymmetric stretching shows a significant shift (up to 2959 cm^−1^) for cells exposed to the 2 Gy dose, while the one related to CH_2_ asymmetric stretching (located at around 2929 cm^−1^ in the average spectrum of control cells) is shifted to a lower wavenumber (2923 cm^−1^) in both 2 and 4 Gy exposed samples. These changes agree with other results reported in the literature [10,11,12,13,15,17,18,20,52,53] for cells exposed to similar or higher ionizing radiation doses. These studies have evidenced significant modifications in lipid content, the presence of lipid peroxidation and saturation processes, and modifications of membrane fluidity that can be related to cell apoptosis which is accompanied by several membrane changes, such as phosphatidylserine exposure, membrane blebbing, and vesicle formation.

In the fingerprint region, it is possible to note a change in the position of one of the Amide I subcomponents related to β-turn structure for 2 Gy exposed samples and of one related to the α-helix structure for 4 Gy exposed samples. Other shifts are also evident in the Amide II and Amide III regions. In particular, a significant upshift is observed for the COO functional group in the Amide II region for irradiated samples (irrespective of the dose). Shifts larger than the spectral resolution are evident for the α-helix (1305 cm^−1^), random coil (1261 cm^−1^), and β-sheet (1225 cm^−1^) components in the case of cells exposed to the 2 Gy dose. For samples exposed to the 4 Gy dose, changes are present for α-helix (the corresponding peaks are moved to 1305 and 1285 cm^−1^) and random coil (to 1262 cm^−1^). The occurrence of more changes in the Amide III region than in the Amide I region agrees with previous results related to the same cells exposed to a proton beam [18]. These shifts can be related to changes in the contributions of the enzymes involved in DNA repair processes ([18,54] and references therein). Modification of protein secondary structure could affect the function of cellular enzymes as well as of structural proteins. Some peaks related to DNA components located in the 1140–950 cm^−1^ region present shifts for samples exposed to 2 and 4 Gy. The shifts in this region can be related to changes in the DNA conformation [55] that are well known to occur in these cells at these doses [19]. More specifically, the contributions due to C-O and PO_2_^−^ symmetric stretching show a wavenumber shift. These changes can be related to alterations in the DNA conformation [55].

In our previous works [17,45], we investigated changes occurring in SH-SY5Y cells by X-ray irradiation at different doses (0, 2, 4, 6, 8, and 10 Gy) fixed immediately after and 24 h after irradiation. The results reported in the aforementioned papers for t_0_ samples irradiated at 2 and 4 Gy also showed changes in FTIR spectra related to modifications in lipids, DNA, protein, and carbohydrates, even if they affected wavenumber shifts for different spectral features. This can be due to the use of a different fitting procedure that has identified a different number of contributions or differences in the cell sample preparation.

### 3.3. Analysis of Infrared Spectra from Cells Fixed 2 h After Irradiation (t_2_ Cells)

The average spectra for samples exposed to 0, 2, and 4 Gy doses of X-rays, and fixed 2 h after irradiation (t_2_ cells), are reported in Figure 2b. As previously said, the results of the deconvolution procedure described in Section Spectra Deconvolution Procedure evidence some wavenumber shifts, even if less numerous than those observed for t_0_ cells. In Table S3, the positions of peaks for t_2_ cells exposed to different doses are reported; shifts in position with respect to the corresponding peak observed in the control sample higher than the spectral resolution available in our experiments are reported in bold characters. In the HWR region, shifts regarding the contributions due to CH_3_ asymmetric and symmetric stretching (located at 2964 and 2876 cm^−1^, respectively) and CH_2_ symmetric stretching (placed at 2655 cm^−1^) are present in the spectra of samples exposed to a 4 Gy dose of X-rays. Also in this case, these shifts are related to changes occurring in lipid contributions to the spectra, as reported in Section 3.2 [10,11,12,13,15,17,18,20,52]. In the fingerprint region, a significant upshift is present for β-sheet contributions (Amide I 1694 cm^−1^ mode and 1199 cm^−1^ mode) when cells are exposed to a 4 Gy dose, suggesting the presence of protein secondary structure modification upon irradiation. Some changes are present in the DNA and carbohydrate region for cells that received a 2 Gy dose. More specifically, the contributions due to C-O and PO_2_^−^ symmetric stretching show a wavenumber shift. These changes can be related to alterations in the DNA conformation [55]. This is in agreement with the well-known effect of X-rays on DNA.

### 3.4. Analysis of Infrared Spectra from Cells Fixed 4 h After Irradiation (t_4_ Cells)

The average spectra for samples that were exposed to irradiation using 0, 2, and 4 Gy doses of X-rays, and fixed 4 h after irradiation (t_4_ cells) are reported in Figure 2c. As in the previous case, they present some wavenumber shifts and some differences in peak absorbance.

In Table S4, the position of peaks for samples exposed to different doses are reported; shifts in position with respect to the corresponding peak observed in the control sample higher than the spectral resolution available in our experiments are reported in bold characters. It is easy to note that the shift present in the HWR for 4 Gy-irradiated samples (CH_2_ asymmetric stretching modes, located at 2927 cm^−1^) indicates that some modifications in membrane lipids that are generally related to membrane fluidity alterations are still present [10,11,12,13,15,17,18,20,52]. In the Amide I spectral range, a shift of the parallel β-sheet component (1631 cm^−1^) can be observed for samples exposed to 2 Gy doses. For the same samples, it is also possible to notice a shift (to 1399 cm^−1^) in the Amide II region related to the COO functional group. In the Amide III region, shifts are present for samples exposed to 2 and 4 Gy. The contribution due to the α-helix subcomponent experiences an upshift (to 1314 cm^−1^) in both cases. For samples exposed to a 4 Gy dose of X-rays, a downshift is also present (to 1289 cm^−1^) for another mode related to the α-helix subcomponent. A significant shift is also observed for contributions located at 1274 cm^−1^ for samples exposed to 2 Gy and related to contribution from Amide III random coil. For samples exposed to 4 Gy, shifts related to DNA contribution are observed for the feature located at 1233 and 1174 cm^−1^. A shift related to DNA (located at 1082 cm^−1^) is also observed for cells exposed to 2 Gy. As said before, these shifts occurring in this wavenumber region are indicative of modifications of DNA conformation [55]. For cells exposed to 4 Gy and fixed 4 h after irradiation, a significant downshift of the COO-stretching mode is also present, and it can be related to changes in lipid contribution in the short wavenumber range.

In all the spectra examined in Section 3.2, Section 3.3 and Section 3.4, changes in the absorbance of some peaks can also be observed due to the exposure to X-rays. These variations will be discussed using the difference spectra, and the ratio values between the intensity of selected peaks [17,18,24,44] in the following sections.

### 3.5. Analysis of Difference Absorbance Spectra

**Figure 3:**
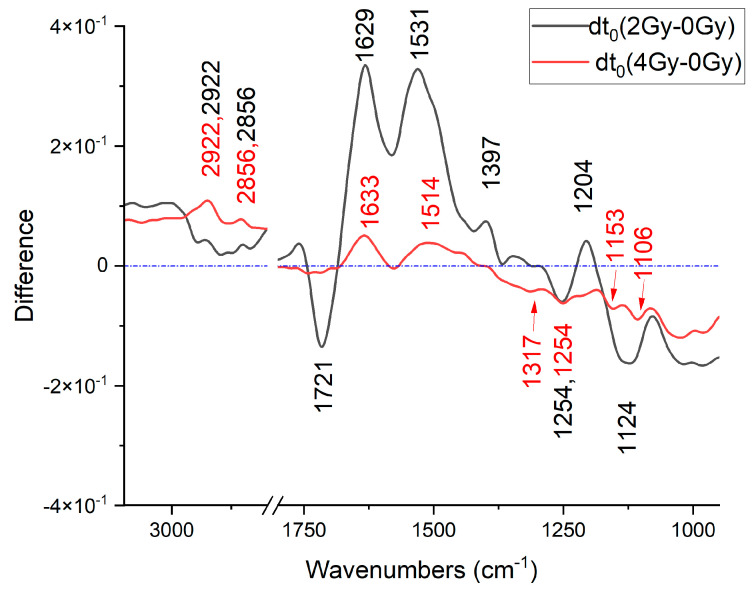
Difference spectra evaluated from absorbance spectra acquired from cells exposed at different radiation doses and fixed immediately after irradiation.

When the spectra are expected to be faintly different, the use of difference absorbance spectra can be useful. In the present case, the difference absorbance spectra are evaluated by considering the average absorbance spectrum for the three different fixation times. The difference absorbance spectra obtained by subtracting the average absorbance spectrum of control sample from the corresponding 2 Gy-irradiated samples and from the corresponding 4 Gy-irradiated samples are considered for t_0_, t_2_, and t_4_ cells, thus obtaining the d_ti_(2 Gy − 0 Gy) and d_ti_(4 Gy − 0 Gy) spectra, where i = 1, 2, and 4, according to the fixation time. The resulting difference absorbance spectra obtained for cells fixed immediately after irradiation (t_0_) are shown in Figure 3, with the positions of the main observed features being labeled.

In the HWR region, relevant positive features are observed at 2922 and 2856 cm^−1^ in both the spectra, indicating that the absorbance at corresponding peaks (FTIR absorbance of modes assigned to CH_2_ and CH_3_ stretching) is higher in the spectra of irradiated samples than in the unirradiated/control samples. The intensity of both the observed features is higher in d_t0_(4 Gy − 0 Gy) than in the d_t0_(2 Gy − 0 Gy) difference spectrum, suggesting that the increase in FTIR absorbance is higher for the lowest dose.

In the fingerprint region, we can observe positive and negative features, i.e., we have both cases: FTIR absorbance of the irradiated sample greater than the absorbance of the control sample at the specific wavenumber (positive features) and FTIR absorbance of the irradiated sample < absorbance of the control sample at the specific wavenumber (negative features). In both the spectra shown in the figure, there is a positive feature at around 1630 cm^−1^ and a negative feature at 1254 cm^−1^. The absorbance at 1531, 1397, and 1204 cm^−1^ is higher for the 2 Gy-irradiated samples than the unirradiated ones, with the d_t0_(2 Gy − 0 Gy) showing positive features. The same curve suggests that the opposite happens at 1721 and 1124 cm^−1^, d_t0_(2 Gy − 0 Gy) showing negative features. In d_t0_(4 Gy − 0 Gy), a positive feature at 1514 cm^−1^ is outlined beside the one at 1630 cm^−1^, along with negative features at 1317, 1153, and 1106 cm^−1^ (and the above-cited feature at 1254 cm^−1^). The intensity of the features observed in the difference spectra shows a specific behavior with the dose. Similar results are obtained by analyzing the same difference spectra calculated for the other samples, namely, t_2_ and t_4_ cells. The results of such an analysis for all the samples are shown in Table 1 in terms of the positions of the features observed with their sign (positive features, +, and negative features, −). As can be seen, a complex pattern of changes is outlined, involving proteins, DNA, and lipids. Clear changes in lipids are observed in irradiated samples at early stages compared to control ones, with the contribution at 2920 cm^−1^ being different in almost all the samples. Very peculiar are the changes in protein contributions in agreement with previous evidence about the occurrence of changes in protein secondary structure upon X-ray irradiation. Changes in DNA-related modes are also observed for all the samples investigated, with the characteristics of the changes depending also on the fixation time, thus showing the occurrence of DNA damage repairing processes.

### 3.6. Ratiometric Analysis

Some of the changes observed in Figure 3 and Table 1 have been confirmed by evaluating the ratios between the selected bands reported in Table 2.

**Figure 4:**
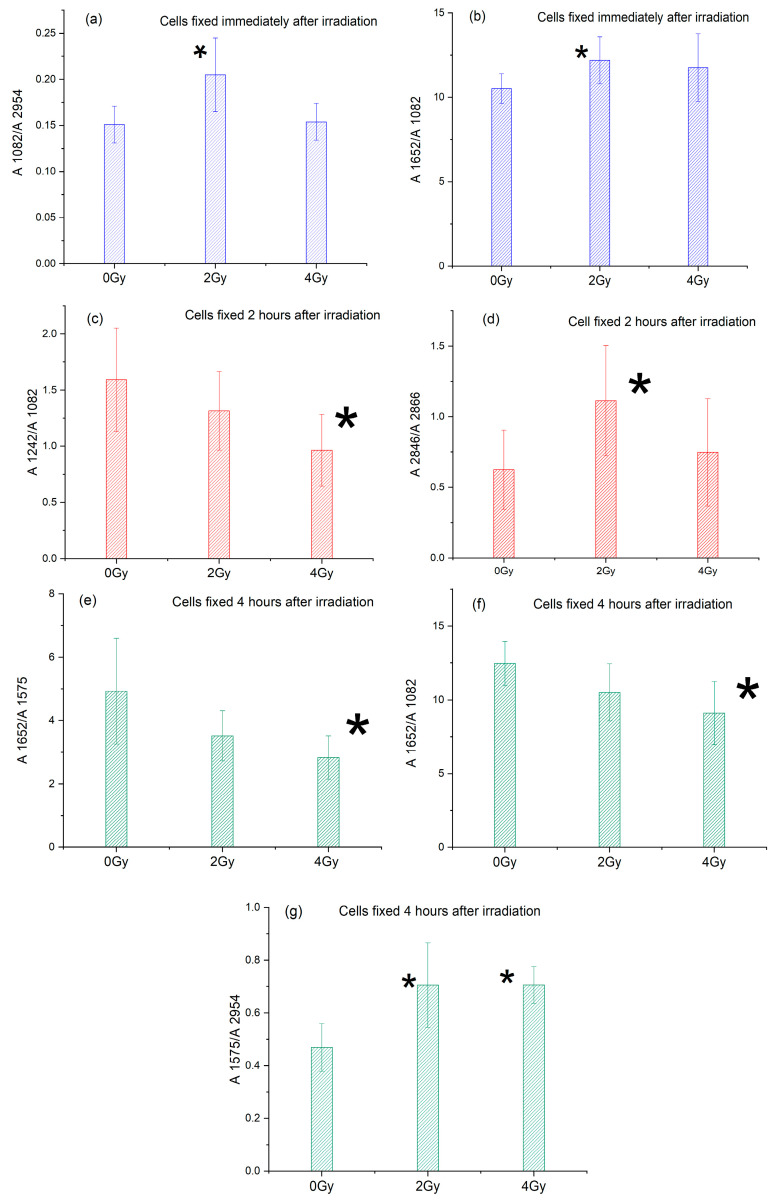
Comparison of the absorbance ratios with dose for cells fixed at different times after irradiation; the ratio variations (mean + SD) are reported. The asterisks indicate when a significant difference occurred at *p* < 0.05.

By comparing the obtained ratio values to the corresponding values of control (0 Gy), cells some changes can be evidenced (see Figure 4 and Table 2). For cells fixed immediately after irradiation, the ratio between the absorbances at 1082 and 2954 cm^−1^ (PP) and the ratio between 1652 and 1082 cm^−1^ absorbances (P/D) obtained for irradiated samples are higher than the ones obtained for the control samples even if the difference is significant only for the 2 Gy-irradiated samples (the values of these ratios are shown in Figure 4a,b), suggesting that 2 Gy X-ray irradiation treatment induces an increase in the protein phosphorylation and in the protein content vs. DNA content. The increase in the protein phosphorylation can be explained by the activation of the DNA damage response (DDR) [56] characterized by a wide network of cellular pathways detecting DNA lesions including single- and double-strand breaks, DNA mismatches, and damaged or inappropriate bases. For example, it is well known that the DDR-related PIKK kinases, such as ATM, ATR, and DNA-PK, upon DNA efficiently phosphorylate the histone variant H2AX in the so-called γH2AX to promote DDR signaling and recruitment of repair machinery as well [57].

For cells fixed 2 h after irradiation, the ratio between the absorbances at 1242 and 1082 cm^−1^ (DM) and the ratio between the absorbances at 2846 and 2866 cm^−1^ (LP) show significant changes compared to the control values, as shown in Figure 4c,d. The DM ratio value for the irradiated samples is lower than for control samples, even if the difference is significant only for the 2 Gy-irradiated samples. The opposite happens for the LP ratio, which is higher for irradiated samples than for control ones; the difference is significant, once again, only for the 2 Gy-irradiated samples. These results suggest a DNA modification and a decrease in lipid peroxidation upon 2 Gy irradiation in t_2_-cells. For cells fixed 4 h after X-ray exposure, the ratio analysis indicates changes in the Amide I/Amide II contributions (obtained by analyzing the ratios between the absorbances at 1652 and 1575 cm^−1^, PR ratio), protein/DNA relative content (information given by the ratios between the absorbances at 1652 and 1082 cm^−1^, P/D ratio), and protein/lipid relative content (P/L ratio between the absorbances at 1575 and 2954 cm^−1^ is estimated), as shown in Figure 4e–g. In particular, the values of the PR and P/D ratios obtained for irradiated samples are lower than for control ones, with the difference being significant only for 4 Gy-irradiated samples. P/L ratio values obtained for irradiated samples are significantly higher than the values for control samples, with the difference significant only for 4 Gy-irradiated cells. These suggest that in t_4_ cells a significant protein rearrangement and a decrease in protein content vs. DNA content are observed upon 4 Gy-irradiation, along with a decrease in lipid content vs. protein content that is evidenced upon both 2 Gy and 4 Gy irradiation. The complex dependence of the observed changes on (fixation) time can be observed by inspecting Table 2, suggesting that there is no monotonic change as a function of time. It can be representative of the complex evolution of single-cell components during the occurrence of the repairing process.

The results of the ratiometric analysis reported here allow us to describe the effects of X-ray irradiation on the SH-SY5Y cells early after irradiation. The effects on DNA of irradiation (with both doses) are confirmed and observed up to 4 h after irradiation, even if changes are observed and associated with the repairing processes enacted by the cells. Notably, thanks to the sensitivity of the μ-FTIR approach to effects on proteins and lipids, this study enabled us to outline an increase in membrane fluidity upon X-ray irradiation (both doses considered here) that lasts at least 2 h after irradiation for the 2 Gy-irradiated sample and lasts up to 4 h after irradiation when the 4 Gy dose is employed; in that case, an increase in protein to lipid content ratio was also observed. The analysis of the effects of irradiation on proteins suggests that 2 Gy X-ray irradiation treatment induces an increase in the protein phosphorylation and in the protein content vs. DNA content along with changes in protein secondary structure still present 4 h after irradiation.

The results discussed here are an additional step in proving that FT-IR has strong potential for application in radiotherapeutic settings as an accurate, rapid, cost-effective, and minimally invasive assessment mode for individual responses to radiotherapy regimens [58]. Previous works have already demonstrated that FT-IR spectroscopy can help monitor radiotherapeutic response in prostate cancer patients [59] and esophageal cancer [60]. Further research efforts are required to overcome translation barriers, and, thus, to allow translation of the FT-IR towards clinical applications in radiobiology and radiotherapy.

## 4. Conclusions

In the present paper, μ-FTIR spectroscopy was adopted for the first time for investigating the changes occurring in the cells exposed to X-rays in the first hours after irradiation. An appropriate deconvolution analysis allowed the identification of all the contributions present in the spectra. Also, the different subcomponent contributions in Amide I, Amide II, and Amide III were identified. The results confirmed that the use of μ-FTIR is useful for sensing the changes induced in cells by ionizing radiation, expanding its use at short times after irradiation. In fact, this approach allowed us to evidence the band shifts induced by X-ray exposure and related to changes in lipid content, the presence of lipid peroxidation and saturation processes, and modifications of membrane fluidity that can be related to cell apoptosis, which is accompanied by several membrane changes, such as phosphatidylserine exposure, membrane blebbing, and vesicle formation. In general, these changes are less evident for the lower doses and longer times considered in the reported investigation. The use of difference spectra confirms these changes, and ratio analysis contributes to a more precise and quantitative description of the processes occurring in the time windows immediately following irradiation. This investigation evidenced that the abovementioned effects were also observed for samples fixed at 24 h after irradiation. Unfortunately, a rigorous comparison with the results previously obtained for the same cell line was not affordable since it is not possible to have samples prepared using exactly the same growing conditions and X-ray treatment previously adopted for the present investigation. As expected from conventional biological assays, significant changes were present. Differently from these assays that give indications only of the processes involving DNA components, μ-FTIR analysis can evidence the changes occurring in all the cellular components. Then the present analysis of the effects of X-ray treatments can contribute to providing an additional piece of information about the early-stage fate of neuroblastoma cells irradiated by using X-rays, clarifying all the processes occurring in the first hours after the irradiation and offering the possibility of a timely check on the efficacy of X-ray treatments. It is worth noting that the different approaches presented here to analyze spectra can be jointly or separately used to monitor radiotherapy effects for personalized radiotherapy treatment.

## Figures and Tables

**Table 1:** Information on relevant peaks observed in the absorbance difference spectra, obtained by subtracting from the average absorbance spectrum of irradiated samples (both for 2 Gy- and 4 Gy-irradiated samples) the average absorbance spectrum of the corresponding control ones for all the investigated t_0_, t_2_, and t_4_ cells. Peaks are reported with their position and in terms of the cellular biomolecules to which they contribute, according to the peak assignment: p = proteins, d = DNA/RNA, l = lipids). Symbols: “+” indicates a positive feature (FTIR absorbance of the irradiated sample > absorbance of the control sample at the specific wavenumber); “−” indicates a negative feature (FTIR absorbance of the irradiated sample < absorbance of the control sample at the specific wavenumber).

Peak Position (cm^−1^)	t_0_ Cells		t_2_ Cells		t_4_ Cells	
	d_t0_(2 Gy − 0 Gy)	d_t0_(4 Gy − 0 Gy)	d_t2_(2 Gy − 0 Gy)	d_t2_(4 Gy − 0 Gy)	d_t4_(2 Gy − 0 Gy)	d_t4_(4 Gy − 0 Gy)
2920–2925 (l)	+	+	−		−	−
2850–2856 (p, l)	+	+			−	
1720 (d)	−					
1695–1700 (p)				−	−	
1650–1655 (p)			−	−		−
1630 (p)	+	+				
1616 (p)			−			
1580–1600 (p)					+	+
1530–1533 (p)	+					−
1514 (p)		+	−		+	
1397 (p)	+					
1317 (p)		−				
1254–1260 (p)	−	−	−	−		
1204 (d, p)	−					
1153 (d)		−				
1124 (d)	−					
1106 (d)		−				+
1086 (d, p)					+	
1030–1050 (d)			−	−	+	+

Legend: d_t0_(2 Gy − 0 Gy) = difference spectrum obtained by subtracting control spectrum to the 2 Gy dose spectrum, t_0_ cells; d_t0_(4 Gy − 0 Gy) = difference spectrum obtained by subtracting control spectrum to the 4 Gy dose spectrum, t_0_ cells; d_t2_(2 Gy − 0 Gy) = difference spectrum obtained by subtracting control spectrum to the 2 Gy dose spectrum, t_2_ cells; d_t2_(4 Gy − 0 Gy) = difference spectrum obtained by subtracting control spectrum to the 4 Gy dose spectrum, t_2_ cells; d_t4_(2 Gy − 0 Gy) = difference spectrum obtained by subtracting control spectrum to the 2 Gy dose spectrum, t_4_ cells; d_t4_(4 Gy − 0 Gy) = difference spectrum obtained by subtracting control spectrum to the 4 Gy dose spectrum, t_4_ cells.

**Table 2:** Values of FTIR absorbance ratios for cells fixed immediately after 2 and 4 h post-irradiation are indicated with the y character. Ax/Ay indicates the ratio between the absorbance of the selected bands [4,17,18,24,44,45]; abbreviation: as = asymmetric, s = symmetric, ν = stretching. Asterisks indicate statistically significant changes (at *p* ≤ 0.05). Pointing-up arrow indicates an increase while pointing-down arrow indicates a decrease.

A_X_/A_Y_	Biomolecular Origin	Indication	Changes Observed for Cells Fixed at t = 0 h		Changes Observed for Cells Fixed at t = 2 h		Changes Observed for Cells Fixed at t = 4 h	
			2 Gy	4 Gy	2 Gy	4 Gy	2 Gy	4 Gy
	***Protein biomarkers***							
A_1652_/A_1575_	Amide I/Amide II-α	Protein rearrangement (PR)	↑	↑	↓	↑	↓	↓ *
A_1082_/A_2954_	PO_2_^−^ s. ν, C-O-P ν/CH_3_ as. ν	Protein phosphorylation (PP)	↑ *	↓	↓	↓	↑	↓
	***DNA biomarkers***							
A_1242_/A_1082_	PO_2_^−^ as. Ν, C-O-P ν/ PO_2_^−^ s. ν, C-O-P ν	DNA modification (DM)	↓	↓	↓	↓ *	↑	↑
	***Lipid biomarkers***							
A_2846_/A_2866_	CH_2_ s. ν/CH_3_ s. ν	Lipid peroxidation (LP)	↑	↑	↑ *	↑	↑	↑
	***Protein/DNA biomarkers***							
A_1652_/A_1082_	Amide I/PO_2_^−^ s. ν	Protein/DNA content (P/D)	↑	↑ *	↑	↓	↓	↓ *
	***Protein/lipid biomarkers***							
A_1575_/A_2954_	Amide II-α/CH_3_ as. ν	Protein/Lipid content (P/L)	↑	↑	↓	↓	↑ *	↑ *

## Data Availability

Data will be available on request.

## Supplementary Materials

The following supporting information can be downloaded at: https://www.mdpi.com/article/10.3390/s24237459/s1, Tables S1–S4.
