# Supplementary material for: Monitoring Biochemical Changes of Neuroblastoma Cells in Early Stages After X-Ray Exposure by Using Fourier-Transform Infrared Spectroscopy
Source: Sensors (Basel). 2024 Nov 22;24(23):7459. doi: 10.3390/s24237459 (PMC11644395; doi:10.3390/s24237459)
Supplement: Supplementary file 1 [file sensors-24-07459-s001.zip › sensors-3255240-supplementary.pdf]

Supplementary Material

Monitoring of Biochemical Changes of Neuroblastoma Cells in Early-Stages after X-ray Exposure by Using Fourier Transform Infrared Spectroscopy

Rosario Esposito[1], Marianna Portaccio[2], Roberta Meschini[3], Ines Delfino[3,4*], Maria Lepore[2]

[1]Dipartimento di Ing. Chimica, dei Materiali e della Produzione Industriale,
Università di Napoli "Federico II" 80125 Napoli, Italy; rosario.esposito2@unina.it
[2]Dipartimento di Medicina Sperimentale, Università della Campania "Luigi Vanvitelli", 80138 Napoli, Italy;
marianna.portaccio@unicampania.it; maria.lepore@unicampania.it
[3] Dipartimento di Scienze Ecologiche e Biologiche, Università della Tuscia, 01100 Viterbo, Italy
[4] INAF- Osservatorio Astronomico di Capodimonte Napoli, Salita Moiariello 16, Napoli, Italy
*Correspondence: delfino@unitus.it

Table S1

FT-IR peaks observed in the spectrum of control cells, with assignments in agreement with the data reported in the literature [S1-S6]; abbreviation: as = asymmetric, s = symmetric, ν = stretching, δ = bending, sc = scissoring, vbr = vibration, a. a. = free amino acids, c = carbohydrates, p = proteins, l = lipids. The indicated position of every peak is the center of the relative pseudo-Voigt function obtained from the deconvolution fit.

Peak Position (cm^{-1}) for control sample fixed at t = 0h	Assignment	Cellular Element
3434	O-H ν	c
3286	Amide A (-N-H ν) O-H ν	p, c
3178	$-NH_3+$ as. ν (a. a.)	p
3069	Amide B (-N-H ν) O-H ν	p, c
2954	CH_3 as. ν	p, l
2929	CH_2 as. ν	l
2866	CH_3 s. ν	p, l
2846	CH_2 s. ν	l
**Amide I region (C=O ν, C-N ν)**		
1694	Antiparallel β-sheet	p
1679	β-turn	P
1674	β-turn	P
1661	α-helix	P
1652	α-helix	P
1644	unordered structures	P
1636	parallel β-sheet	P
1624	parallel β-sheet	P
1614	antiparallel β-sheet	P
**Amide II region (C-N ν, N-H δ)**		
1575	Amide II (C-N ν, C-NH δ, α-helix)	
1539	Amide II (C-N ν, C-NH δ, β-sheets)	p
1453	CH_3 as. δ, CH2 sc.	p, l
1389	COO− s. ν	p
**Amide III region**		
1314	α-helix	p
1294	α-helix	p
1281	random coil	p
1256	random coil	p
1242	PO^{2-} as. ν	DNA, l, p

1231	PO^{2-} as. ν β-sheet	DNA, p
1220	β-sheet	DNA, p
1204	C-H ring δ, β-sheet	DNA, p
1192	β-sheet	p
1175	sugar-phosphate backbone vbr	DNA
1161	CO-O-C s. as. ν	l
1148	ribose C-O ν	DNA
1119	ν -C-O	DNA
1104	P-O-C s. ν	DNA
1082	PO^{2-} s. ν C-O-P ν	DNA, p
1053	ν -C-O	DNA
972	PO^{4-} s. ν C-0 ν,<br>C=C ν (a.a.)	DNA, p

Table S2

Average FTIR peaks position for control and samples treated with the different doses of X-rays fixed immediately after irradiation (t_0 cells). Bold values indicate shifts greater than the spectral resolution of the instrument 4 cm^{-1}.

Peak position (cm^{-1}) for control samples fixed at t = 0h	Peak position (cm^{-1}) for 2Gy irradiated samples fixed at t = 0h	Peak position(cm^{-1}) for 4Gy irradiated samples fixed at t = 0h
3434	**3443**	3433
3286	3289	3286
3178	3178	**3170**
3069	**3059**	**3068**
2954	**2959**	2954
2929	**2923**	**2923**
2866	2866	2866
2846	2846	2846
	**Amide I region**	
1694	1694	1694
1679	1680	1680
1674	**1680**	1674
1661	1664	**1666**
1652	1654	1653
1644	1648	1644
1636	1638	1637
1624	1626	1624
1614	1614	1614
	**Amide II region**	
1575	1575	1575
1539	1539	1539
1453	1450	1453
1389	**1398**	**1394**
	**Amide III region**	
1314	**1305**	**1305**
1294	1295	**1285**
1281	1281	1283
1256	**1261**	**1262**
1242	1239	1242
1231	**1225**	1229
1220	1216	1220
1204	1204	1206
1192	1193	1191
1175	1175	1173
1161	1161	1161
1148	**1138**	**1142**

1119	**1129**	**1126**
1104	1105	1103
1082	1081	1081
1053	1053	**1059**
972	**958**	**965**

Table S3

Average FTIR peaks position for control and samples treated with the different doses of X-rays fixed 2 hours after irradiation (t_2 cells). Bold values indicate shifts greater than the spectral resolution of the instrument 4 cm^{-1}.

Peak position (cm^{-1}) for control samples fixed at t = 2h	Peak position (cm^{-1}) for 2Gy irradiated samples fixed at t = 2h	Peak position (cm^{-1}) for 4Gy irradiated samples fixed at t = 2h
3441	3443	**3433**
3287	3286	3286
3178	3178	3178
3061	3059	3061
2959	2960	**2964**
2923	2922	**2929**
2866	2866	**2876**
2846	2846	**2855**
	**Amide I region**	
1685	1686	**1694**
1679	1679	1676
1674	1673	1674
1664	1661	1667
1656	1652	1655
1644	1644	1642
1634	1636	1632
1627	1624	1624
1614	1614	1614
1612	1614	1614
	**Amide II region**	
1576	1575	1575
1539	1539	1539
1453	1453	1453
1394	1396	1395
	**Amide III region**	
1312	1312	1314
1295	1295	1295
1283	1283	1283
1261	1265	1263
1239	1242	1240
1225	**1230**	**1231**
1214	1218	1218
1202	1203	1203
1194	1190	**1199**
1168	1169	1166
1162	**1169**	1166

1140	**1148**	1141
1120	**1126**	1121
1101	1100	1098
1084	**1090**	1081
1062	**1057**	**1055**
962	965	962

Table S4

Average FTIR peaks position for control and samples treated with the different doses of X-rays fixed 4 hours after irradiation (t_4 cells). Bold values indicate shifts greater than the spectral resolution of the instrument 4 cm^{-1}.

Peak position (cm^{-1}) for control samples fixed at t = 4h	Peak position (cm^{-1}) for 2Gy irradiated samples fixed at t = 4h	Peak position (cm^{-1}) for 4Gy irradiated samples fixed at t = 4h
3443	3443	**3438**
3290	3289	3287
3178	3178	3178
3059	3059	**3069**
2958	2958	2960
2922	2922	**2927**
2866	2866	2866
2846	2846	2846
	**Amide I region**	
1694	1694	1694
1680	1680	1680
1674	1673	1674
1664	1662	1665
1652	1653	1652
1644	1644	1643
1636	1635	1636
1624	**1631**	1627
1614	1614	1615
	**Amide II region**	
1575	1575	1575
1539	1539	1539
1453	1453	1453
1393	**1399**	1394
	**Amide III region**	
1308	**1314**	**1314**
1295	1294	**1289**
1282	**1274**	1283
1263	1266	1259
1242	1240	**1233**
1231	1231	1233
1219	1218	1216
1204	1200	1203
1194	**1200**	1194
1167	1171	**1161**
1169	1171	**1174**
1138	1139	1141

1123	1127	1127
1101	1099	1102
1089	**1082**	1086
1053	1054	1053
958	959	958

REFERENCES

S1. Gault, N.; Lefaix, J.L. Infrared microspectroscopic characteristics of radiation-induced apoptosis in human lymphocytes. Radiat. Res. 2003, 160, 238–250.

S2. Gault, N.; Rigaud, O.; Poncy, J.L.; Lefaix, J.L. Infrared microspectroscopy study of γ-irradiated and H2O2-treated human cells. Int. J. Radiat. Biol. 2005, 81, 767–779.

S3. Meade, A.; Clarke, C.; Byrne, H.; Lyng, F. Fourier transform infrared microspectroscopy and multivariate methods for radiobiological dosimetry. Radiat. Res. 2010, 173, 225–237

S4. Lipiec, E.; Kowalska, J.; Lekki, J.; Wiechec, A.; Kwiatek, W.M. FT-IR Microspectroscopy in Studies of DNA Damage Induced by Proton Microbeam in Single PC-3 Cells. Acta Phys. Pol. A 2012, 121, 506–509.

S5. Barth, A. Infrared spectroscopy of proteins. Biochim. Biophys. Acta 2007, 1767, 1073–1101.

S6. Barraza-Garza, G.; Castillo-Michel, H.; De La Rosa, L.A.; Martinez-Martinez, A.; Pérez-León, J.A.; Cotte, M.; Alvarez-Parrilla, E. Infrared spectroscopy as a tool to study the antioxidant activity of polyphenolic compounds in isolated rat enterocytes. Oxid Med. Cell Longev. 2016, 9245150
